# Supplementary material for: Ciliate Microtubule Diversities: Insights from the EFBTU3 Tubulin in the Antarctic Ciliate Euplotes focardii
Source: Microorganisms. 2022 Dec 6;10(12):2415. doi: 10.3390/microorganisms10122415 (PMC9784925; doi:10.3390/microorganisms10122415)
Supplement: Supplementary file 1 [file microorganisms-10-02415-s001.zip › microorganisms-2041260-supplementary.pdf]

microorganisms

Supplementary Material

Table S1. List of the primers.

Name		Sequence	Amplicon Length
EFBTU2sil	forward primer	5′-GCCATCCAAGAAATGTTCAAGA-3′	201 bp
	reverse primer	5′-TTCATCGTCGAATTCTCCTTCT-3′	
EFBTU3sil	forward primer	5′-CAGACATGGAAGATACTATA-3′	406 bp
	reverse primer	5′-TTCATCATCAAATTCTCCTTC-3′	
Beta1_qRT-PCR	forward primer	5′-GATCGTACATATCCAAGCAGGT-3′	136 bp
	reverse primer	5′-ATTGATTCTCTCCAATTGAAGATCG-3′	
Beta2_qRT-PCR	forward primer	5′-CATTCAAGCAGGACAGTGTGG-3′	146 bp
	reverse primer	5′-TGGCTTCGTTGTAGTAAACGTTA-3′	
Beta3_qRT-PCR	forward primer	5′-GAGTCAGAAGAATGCGATTGTC-3′	199 bp
	reverse primer	5′-TGGAAAGGGTGGTATTGTATGG-3′	
SSUrRNA_qRT-PCR	forward primer	5′-GATTACGTCCCTGCCCTTTGT-3′	141 bp
	reverse primer	5′-ACCTTGTTACGACTTCTCCTTCC-3′	

Table S2. Values of standard curves for each primer set.

Primer Set	Efficiency (%)	Slope (R^2)	y-Intercept
Beta1_qRT-PCR	100.1	0.993	20,544
Beta2_qRT-PCR	100.4	0.974	21,079
Beta3_qRT-PCR	100.2	0.995	20,232
SSUrRNA_qRT-PCR	97.9	0.992	19,428

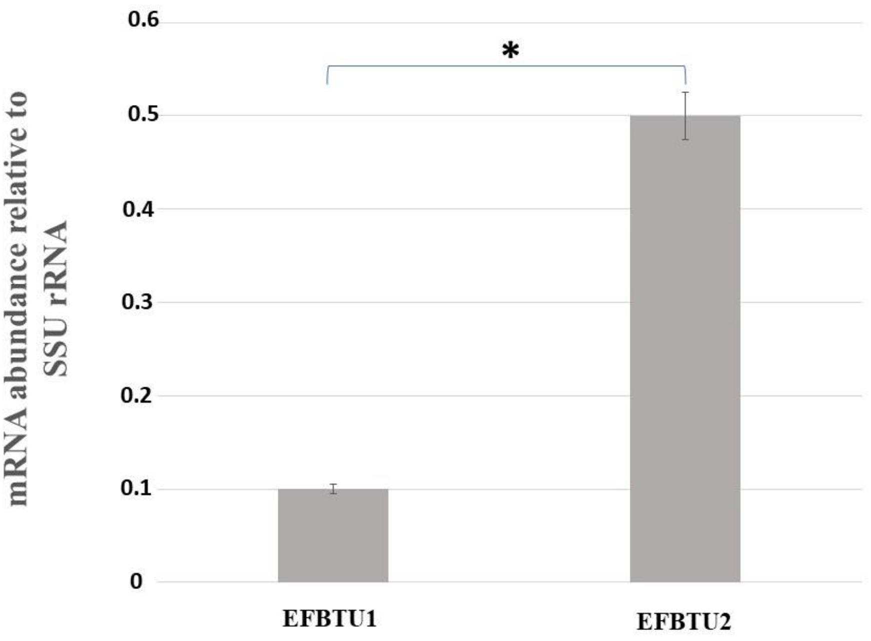

Figure S1. EFBTU1 and EFBTU2 transcription level analyzed by qRT-PCR. Total RNA was extracted from cells at the end of the log phase. To distinguish the transcription level of the two genes, we used oligonucleotides complementary to portions of the coding regions with different codon usage. Data are reported as the mean of three experiments, referred to the expression level of the SSUrRNA,

used as housekeeping gene. The significance of the different transcription level of the two genes is supported by the estimated *p*-value < 0,05 indicated by the *. Beta1_qRT-PCR, Beta2_qRT-PCR primers and SSUrRNA_qRT-PCR primer sets are listed in Table S1, the values of the standard curves are listed in Table S2.

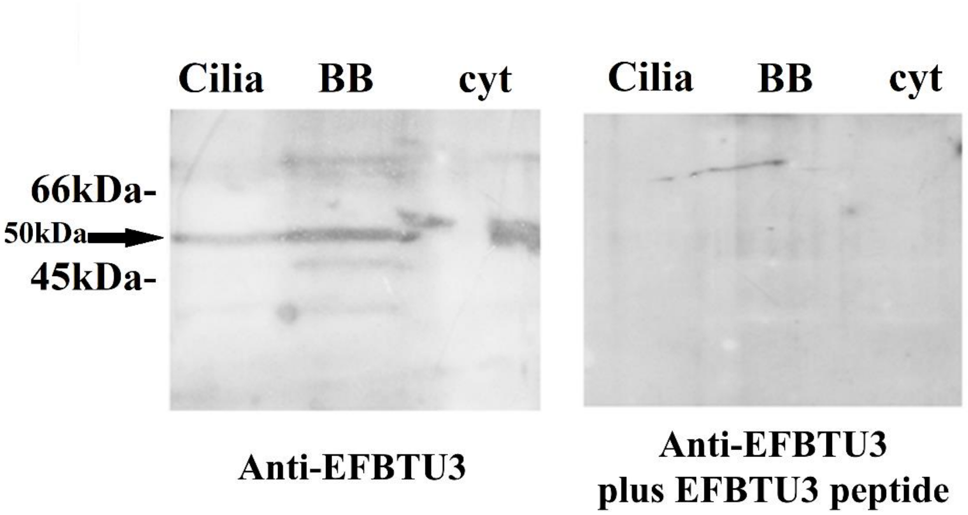

Figure S2. Immunodetection of EFBTU3 in *E. focardii* cellular subfractions by western blot. Cellular subfractions are enriched in basal bodies (BB), cilia and cytoplasmic microtubules. The blots were incubated with anti-EFBTU3 antibodies in presence (right blot) or absence (left blot) of EFBTU3 peptide. The 50 kDa EFBTU3 protein is indicated by the arrow. The molecular weights of protein standards are indicated on the left. The fainter bands are interpreted as products of non-specific recognition by the polyclonal antibodies.

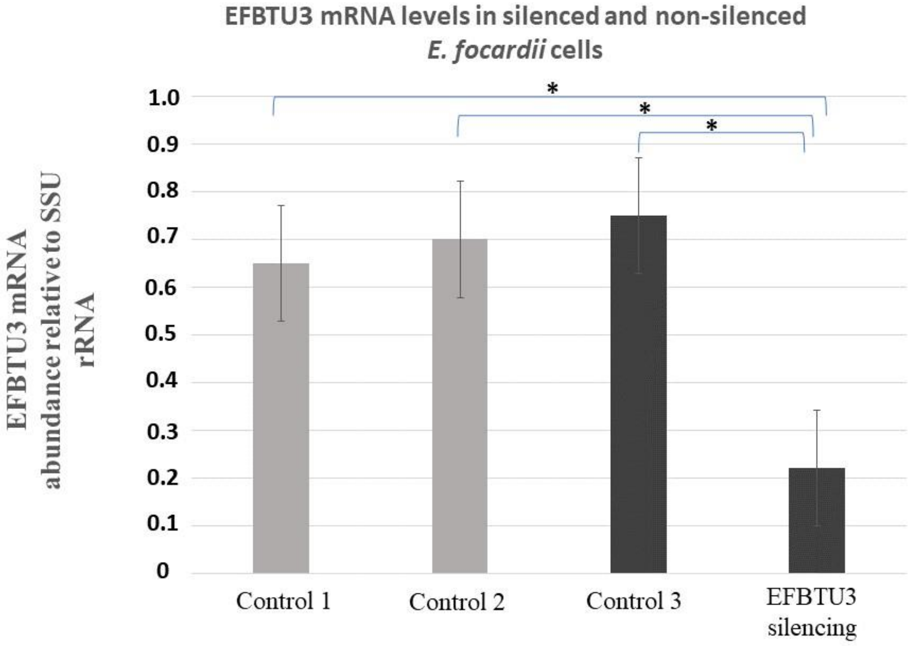

Figure S3. EFBTU3 mRNA levels analyzed by qRT-PCR from *E. focardii* cell cultures fed with non-induced bacteria containing empty L4440 and EFBTU3-L4440 vectors (control 1 and control 2, respectively), and IPTG-induced bacteria containing empty L4440 and EFBTU3-L4440 vectors (control 3 and EFBTU3 silencing, respectively). In control 1, 2 and 3, equal levels of EFBTU3 transcripts are detected, while in EFBTU3 silencing sample there is a strong decrease (*p*-value < 0.05, indicated by

the *) in the EFBTU3 transcripts, as expected upon siRNA-based gene silencing. Data are reported as the mean of three experiments.

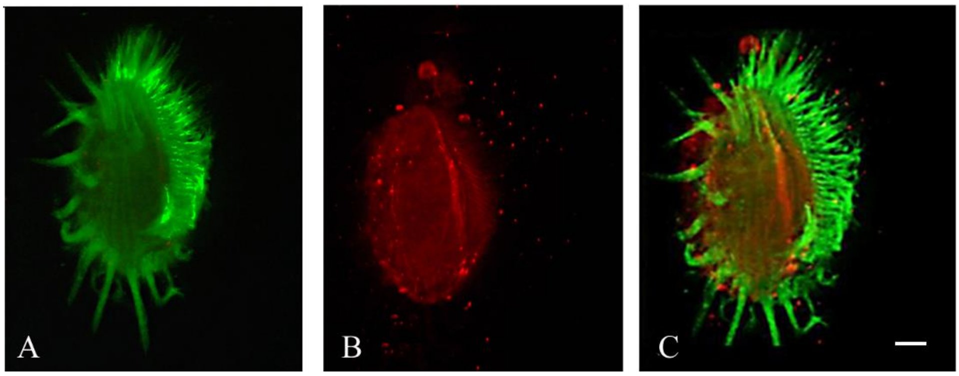

Figure S4. Immunofluorescence microscopic images of one *E. focardii* cell after EFBTU3 gene silencing. The cell was co-stained with the commercial anti-β-tubulin antibodies (green, **A** and **C**) and anti- EFBTU3 antibodies (red, **B** and **C**). As a result of the EFBTU3 silencing the EFBTU3 staining appears not specific. Scale bar corresponds to 10 µm.

Table S3. Percentages of cells showing complete, partial, or no regeneration of cilia for samples collected after 4 and 24 hours from deciliation.

	**Time from Deciliation**	
	**4 h**	**24 h**
Complete cilia regeneration	0%	8%
Partial cilia regeneration	16%	34%
No cilia regeneration	84%	58%

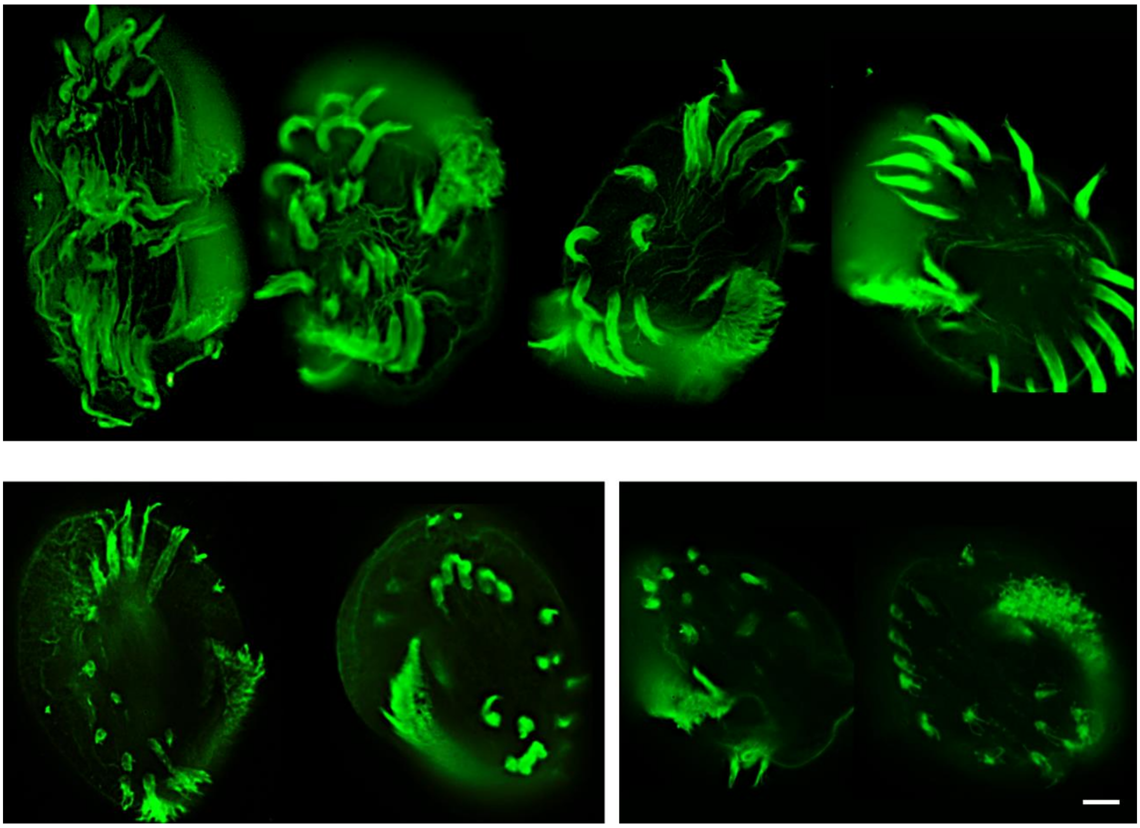

Figure S5. Immunofluorescence microscopic images of *E. focardii* cells collected after 24 hours from deciliation and incubated with commercial anti-β-tubulin antibodies. The upper panel shows control cells (fed with IPTG-induced bacteria containing empty L4440 vector) with ciliary structures completely regenerated. The lower panels show silenced cells (fed with IPTG-induced bacteria containing EFBTU3-L4440 vector) with partially regenerated cilia (left) and no regenerated cilia (right). Scale bar corresponds to 10 μm.

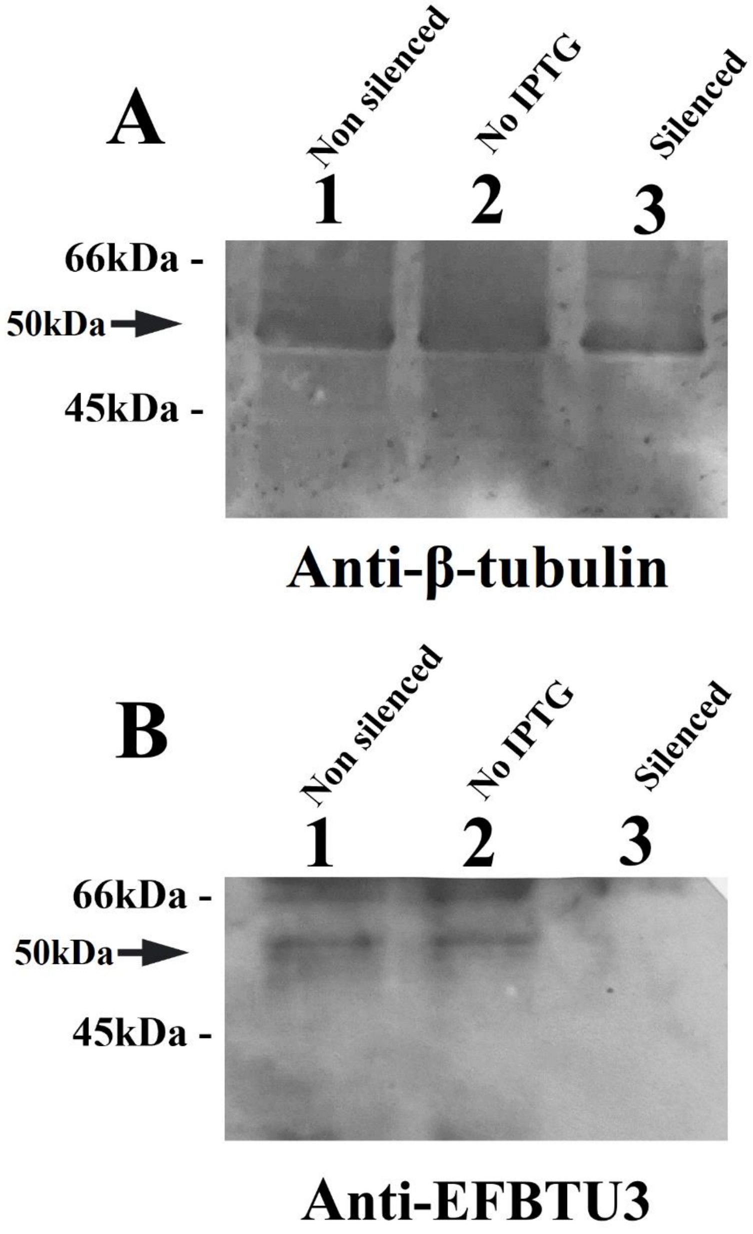

Figure S6. Immunodetection by western blot of total protein extracts obtained 24 after deciliation from EFBTU3-silenced and non-silenced *E. focardii* cells. The same membrane was first incubated with monoclonal anti-β-tubulin antibodies (A) and, after stripping, with polyclonal EFBTU3 antibodies (B). The 50 kDa EFBTU3 protein is indicated by the arrow. In both panels, lanes 1, 2 are

protein samples from non-silenced cells, while in lane 3 proteins from EFBTU3-silenced cells were loaded. The arrows indicate the β-tubulin protein pool (A) and EFTBTU3 (B). The molecular weights of protein standards are indicated on the left side of both panels.
